# Overcoming Systemic Barriers Preventing Healthy Urban Development in the UK: Main Findings from Interviewing Senior Decision-Makers During a 3-Year Planetary Health Pilot

**DOI:** 10.1007/s11524-021-00537-y

**Published:** 2021-05-03

**Authors:** Daniel Black, Paul Pilkington, Ben Williams, Janet Ige, Emily Prestwood, Alistair Hunt, Eleanor Eaton, Gabriel Scally

**Affiliations:** 1 Daniel Black + Associates | db+a, University of Bristol, Bristol, UK; 2 UWE Bristol, Bristol, UK; 3 University of Birmingham, Birmingham, UK; 4 University of Bath, Bath, UK; 5 University of Bristol, Bristol, UK; 6 Gabriel Scally Public Health Associates, Bristol, UK

**Keywords:** Urban development, Planetary health, Elite interviewing, Upstream decision-making, Complex systems

## Abstract

This paper sets out the main findings from two rounds of interviews with senior representatives from the UK’s urban development industry: the third and final phase of a 3-year pilot, Moving Health Upstream in Urban Development’ (UPSTREAM). The project had two primary aims: firstly, to attempt to value economically the health cost-benefits associated with the quality of urban environments and, secondly, to interview those in control of urban development in the UK in order to reveal the potential barriers to, and opportunities for, the creation of healthy urban environments, including their views on the use of economic valuation of (planetary) health outcomes. Much is known about the ‘downstream’ impact of urban environments on human and planetary health and about how to design and plan healthy towns and cities (‘midstream’), but we understand relatively little about how health can be factored in at key governance tipping points further ‘upstream’, particularly within dominant private sector areas of control (e.g. land, finance, delivery) at sub-national level. Our findings suggest that both public and private sector appeared well aware of the major health challenges posed by poor-quality urban environments. Yet they also recognized that health is not factored adequately into the urban planning process, and there was considerable support for greater use of non-market economic valuation to help improve decision-making. There was no silver bullet however: 110 barriers and 76 opportunities were identified across a highly complex range of systems, actors and processes, including many possible points of targeted intervention for economic valuation. Eight main themes were identified as key areas for discussion and future focus. This findings paper is the second of two on this phase of the project: the first sets out the rationale, approach and methodological lessons learned.

**Supplementary Information:**

The online version contains supplementary material available at 10.1007/s11524-021-00537-y.

## Introduction

There is now significant evidence linking the quality of urban environments to human and planetary health, and there are increasing calls to shift our focus to factors upstream, in particular to the role of the private sector and the ‘commercial determinants of health’; these positions we set out in detail in linked papers [1–3]. This paper sets out the main findings from two rounds of 30 interviews with senior representatives from the UK’s urban development industry—the third and final phase of a three-year pilot, Moving Health Upstream in Urban Development’ (UPSTREAM)—discusses their implications and points to where further research is needed. The findings presented here draw on 69 pages of field notes and 384 pages of transcriptions. The data resulted from the second of the pilot study’s two main aims, which were to:

1. Develop the use of economic valuation in understanding the quality of the urban environment and its measurable impact on human and planetary health [4].
2. Investigate, through face to face engagement with those in control of the urban development in the UK, the main barriers and opportunities for creating healthy urban environments.

The first round of interviews consisted of a broad exploration of factors, starting with 13 thematic areas and associated probes, which focused on interviewee (and organisational) roles and responsibilities and their views on the main barriers and opportunities. In the second, we first sought interviewees’ responses to headline findings from the economic valuation of urban-health evidence produced in the first two phases of the project, before investigating more deeply five themes that interviewees had flagged repeatedly in the first round (the rationale for the approach, and methodological lessons learned, are provided in our sister manuscript [5].).

In addition to their understanding of urban health and their views on economic valuation, interviewees identified 110 barriers and 76 potential opportunities that may be impacting on the development of healthy urban environments. We collated these under eight main themes: (i) valuation, (ii) finance, (iii) land, (iv) partnership, (v) politics, (vi) public realm, (vii) policy, and (viii) capacity. A final cross-cutting theme—‘intractable challenges’—covers a range of issues that appear to be either difficult or impossible to solve.

The findings and associated quotations (see supplementary material) present a summary of findings due both to the amount of data and the complexity of the systems investigated. This limited presentation may suggest a weakness given that in some instances the findings appear cursory, but to arrive at that conclusion would, we feel, miss a central point of this study. Each of these themes are areas of deep specialisation in their own right, so the value of this broad, system-wide approach is not in the depth of disciplinary investigation, but in its breadth, and the opportunity to draw together multiple strands from multiple systems, thereby ‘balancing complexity and the reduction of that complexity’ [6]. Though there is not the space within this paper to unpick each insight gleaned, we believe there are significant findings throughout the main themes, and we discuss these briefly at the end of the document.

The aim of this paper therefore is to provide—through the lens of human and planetary health—a preliminary, system-wide insight into a number of overlapping disciplines involved in UK urban planning and development.

## Urban Development Agencies’ Understanding of Urban Health

‘Most are obvious, but dementia surprising’.

While interviewees were unaware of one or two more nuanced links—the impact of road traffic noise on the cost of treating child conduct disorder generated most surprise, and the link between air pollution and dementia was a surprise to some—the majority of urban health challenges are well known to decision-makers, including air pollution, excessive car use, obesogenic food environments, mental health, and the need for access to nature.

## Valuation

‘…the negative impacts… how are they captured…they’re not at the moment!’

All bar one of the interviewees agreed that health is not being taken into account sufficiently when making decisions in urban development, and they would support the types of valuation mechanisms presented to them by UPSTREAM team: they (a) understood the uncertainties inherent in this type of approach, (b) were less interested in it as a tool for precise market cost-benefit appraisal and (c) more interested in it as an enabler for prioritising issues, understanding orders of magnitude and communicating value.

Yet there was a notable scepticism, particularly from the private sector, at being able to factor in these external costs. It was pointed out that existing valuation mechanisms (e.g. RICS ‘Red Book’, a global standard in asset valuation) were not designed for these ‘intangibles’. Interviewees acknowledged that valuation mechanisms are open to misuse, underlined the need for independent validation, tools, guidance and communication and a level playing field. Despite these reservations, however, several of the same private sector interviewees’ companies had already commissioned consultancies to undertake valuations of this kind to demonstrate the social value benefits of their developments.

**Fig. 1:**
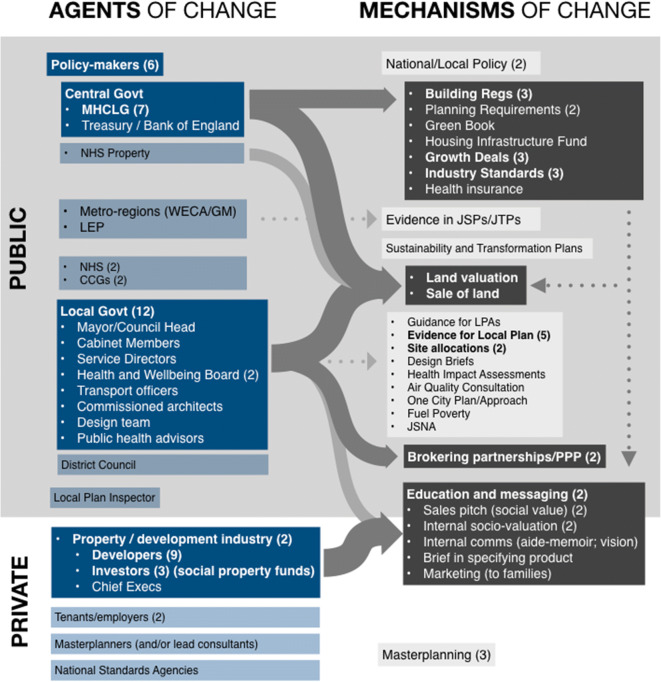
Illustration showing interviewee suggestions for where non-market economic valuation might be used to factor health outcomes into the urban development system, and which agent is responsible. The numbers given in brackets show the recorded number of interviewees that suggested those actors or mechanisms. The colour of the boxes and the size of the arrows illustrate the combined interviewees’ views on the primary areas of control. Acronyms are described at the end of the manuscript. Acronyms are expanded below

Interviewees suggested a very wide range of leverage points at which this type of valuation might be fed into the urban development system, both private and public sector and at national and local level. Figure 1 lists the actors and mechanisms suggested by interviewees, which we have grouped according to their sector, organisation and tier of governance.

## Finance

‘…making a profit, but not profiteering...’

Though a small number of private sector interviewees contested that short-term financial horizons necessarily resulted in lower quality (i.e. corporate reputation, differentiation of higher quality ‘products’), by and large they were recognized as a major issue by both the public and private interviewees (i.e. shareholder expectations and six-monthly reporting; sale of public assets for short-term gain; durability versus cost of materials). Multiple interviewees flagged the perceived benefit of patient capital (e.g. pension funds) as an antidote to financial short-termism, but there were three clear obstacles given: (a) they have to operate at large scale; (b) they are not all long term (often 5–7 years); and (c) they are reliant on long-term revenue (i.e. not market-sale housing, which makes up the large majority of new and existing housing in the UK). In London and other major cities, there is also a substantial reliance on foreign investment, particularly from the USA and China, to provide longer-term financing. Despite the economic downturn since 2008, there was a broad sense that money is available as long as the conditions are otherwise right. A further challenge presented was that higher density development requires greater up-front investment, higher profit margins, and a cash flow model that is markedly different from plot-by-plot sales.

The role of finance in provision of community infrastructure (e.g. public transport, local amenities and public realm) was raised repeatedly, and public sector investment was seen as crucial for the forward funding of infrastructure (e.g. paying up front for city tram lines, which won’t immediately recoup their losses), not least due to their ability to borrow more cheaply. It was also acknowledged however that local government is constrained in its lending, borrowing or otherwise take on risk, which shifts responsibility to the private sector and is more expensive.

## Land

‘…our business, a fundamental part of it is land acquisition. We can’t do anything without land…it’s very dangerous when you try and intervene in things like (land value capture)…’

Control of land was seen as critical by all parties, though one interviewee asserted that ‘clear path to control’—i.e. sites may be controlled through securing of ‘options’ agreements, or other partnership arrangements—can be as effective as having it under one ownership. There was an acknowledgement of the increasing appetite within some local authorities, particularly the larger cities, for proactively using their land as equity. There was broad acknowledgement too that, without land ownership, the public sector had very limited control over the quality of development, though some private sector interviewees contested that necessity.

There was a widespread acknowledgement that decisions around land disposal either prioritize or solely consider commercial aspects and that the mechanisms for considering whether sites are appropriate or desirable for development do not consider health implications beyond basic minimums, as well as a specific query from interviewees about whether public sector agencies, NHS Property in particular, should be more actively engaged in this area.

When asked about the widely held perceptions of ‘land banking’—the buying and holding of land for later development and disposal—it was readily acknowledged and coherently justified that land is acquired strategically as an integral and natural part of volume housing delivery [7–9]. Only one interviewee—across both public and private sector interviewees—was against the notion of ‘land value capture’ (the mechanism for capturing the increase in value that often goes to the private sector but arises from public investment in infrastructure, increased prosperity and/or the granting of planning permission) [10]. The language used by the one interviewee in opposition was emphatic however, revealing a significant potential tension. More fundamentally perhaps, one of those same interviewees from the private sector who supported the notion of land value capture in principle also pointed out that this was just another mechanism of taxation and queried why it was fair that landowners should be taxed and not, for example, the global technology firms. It was acknowledged that clear guidance (on land value capture) may be needed and it would require national leadership.

## Partnership

‘You’ve got to understand what you’re putting in to the pot, and be able to value that. If it’s only 10% of the partnership, then you only get 10% of the control’.

The theme of partnership was notable for two main reasons: firstly, for the level of importance given to it by interviewees (and how it underpins other core areas of risk, land and finance) and, secondly, for the multiplicity of characteristics and mechanisms that were suggested make up successful partnership: e.g. trust, shared values and vision, track record and parity of control. In other words, there was a clear lack of shared definition of what is meant by the term ‘partnership’.

Several interviewees highlighted the need to share value propositions, not just with regard financing, but also social value, procurement processes and even land receipt, and to develop shared vision statements and associated legal mechanisms to reduce risk and ensure clarity. On the role of the private sector, it was acknowledged that there is a wide range in quality between different private sector developers that the quality of the developer makes a significant difference to quality of environment and that partnering with higher quality developers was not always possible. Conversely, it was underlined that the onus is not just on the private sector, but the track record of local authorities, albeit with the acknowledgement that local government currently have very little resource. It was also pointed out that, while housing associations have ‘no shareholders to worry about’, they also are likely also to have in place limited risk monitoring. This line of questioning was linked to the new and increased competition between social housing providers and volume-house builders in market sale development.

## Politics, Education and Communication

‘Politicians benefit from announcing a policy; they don’t always benefit from its delivery…elections…drive a lot of extremely short-term decision-making...’

Shifts in political administration were acknowledged as being problematic, but remarkably little and with the same resigned acceptance as financial short-termism. Only one interviewee felt strongly about short-term politics being a barrier, while another underlined the dominance of the agenda-setting. The role of education was alluded to time and again, but indirectly, and in different ways (i.e. house-buyers realising the benefits of healthier buildings (and being willing to pay for it); mortgage lenders understanding the difference in quality of developments; the electorate in supporting the political administration; professional expertise in delivery). Overall, there was a sense conveyed by interviewees of the primacy of agenda-setting, juxtaposed strikingly against an absence of ideas about how to effect change.

## Public Realm

‘…we don’t retain an ownership in a development…our business is about developing and selling houses and moving on…’

Though ostensibly a minor issue (when compared with major controlling mechanisms such as investment and politics), the importance and cost of maintaining the public realm were cited frequently as a key challenge in the creation of healthy urban development. Of the two options given—adoption by a public body or private sector management (funded by service charges taken from residents)—the latter was seen as the only viable solution given government cuts to public services, yet developers often do not want to retain ownership, and interviewees’ responses suggest that management companies are neither well established nor well regulated.

## Policy, Legislation and Regulation

‘…there was a Planning Guidance (PPG3) that set minimum densities…now if you visit those developments, they are a complete nightmare...’

Given the fundamental role of policy, legislation, and regulation in urban development, it is remarkable that there was relatively little discussion of these ubiquitous controlling mechanisms. Questions were raised about the relative merits of government intervention to support healthier development and for various reasons, not only its efficacy but also the (potentially significant) gap between policy and implementation. Developers were not averse to government intervention, but naturally expected fairness. The over-riding impression given was that change was not anticipated from government policy or legislation.

## Capacity and Resource

‘There’s no doubt there’s a lack of resource in a lot of authorities…it’s not a big issue for us’.

A current and significant lack of resource and capacity within the public sector was clearly articulated in the interviews (i.e. capital budgets, number of staff, level of expertise), not just by the public sector interviewees (e.g. low salaries, lack of opportunity, lack of resource to create change) but also by the private sector (e.g. protracted negotiations, delays). This is a well-reported issue, particularly since the 2008 financial crash, but a more nuanced finding is that these cuts seem to have affected the private sector relatively little.

## Intractable Challenges

A number of issues raised by interviewees appear to be either difficult or impossible to solve. Some align to the themes identified (Box 1), but a similar number sit outside under their own categories: conflict in (understanding of) best practice, conflicting priorities, speculation vs risk aversion. public vs private. health ‘premium’ vs affordability and scale vs feasibility (Box 2).

Box 1 Intractable challenges within main themes identified

**Table:** 

**Finance** • Private sector lending is more expensive and readily available; public funding is cheaper, but often unavailable • Public transport infrastructure takes time to develop and funding is rarely available, which adds pressure to allow parking and increase car use **Land**: • Land is cheaper in areas of low demand yet development is unviable and vice versa **Politics**: • Mayoral system offers greater powers, but success depends on the direction of travel and suffers from the usual political swings • Prioritisation of issues is critical, but there is no clear strategy of governance towards long-term health outcomes **Policy**: • ‘Five-year land supply’ policy is meant to free up land but naturally encourages strategic holding • Certain policies (e.g. PPG3) have been designed to improve quality of built environment, but misalignment with implementation has resulted in worse quality urban environments **Capacity**: • Significant potential for planning to affect health, but in practice this is very difficult to achieve due to lack of capacity and resource • The private sector expect initiatives in urban health to come from the public sector, but the latter lacks resource at local level and prioritisation of longer-term issues at national level	

Box 2 Additional intractable challenges

**Table:** 

**Conflict in best practice:** • Despite long-standing best practice guidance on urban design/planning, there is still confusion about trade-offs, e.g.: -.Urban (access to jobs, amenities, community) vs. sub-urban (access to nature, tranquility and clean air) - Socio-economic necessity (e.g. use of car to access jobs) vs active travel (e.g. on rural/suburban greenfield sites, cycling seen as leisure-only activity, not as a mode of travel) **Conflicting priorities**: • Demands different (e.g. consumer/convenience vs government/politics/economics vs scientist/health/climate) • Certain trade-offs irreconcilable— prioritisation needed (e.g. sports pitches vs woodland) **Speculation vs risk aversion**: • Developers specialize in speculation, but shareholders require low risk (i.e. 10% ROI; ‘would go out of business at 15% profit’) • ‘Intensive systems’ of risk management controls therefore crucial, but also severely constraining **Public vs private**: • Private rental sector has the coldest homes, which Councils are not able to remedy **Health ‘premium’ vs affordability**: • Private sector expect purchasers to pay for health premium (i.e. additional cost of higher quality development), yet house prices/affordability a constant challenge and priority	

## Discussion

The comprehensive support from interviewees for our approach to non-market valuation is a strong mandate for the development and testing of new methods in this area, albeit with significant caveats regarding validation and the ensuring of a level playing field.

The use of economic valuation approaches in measuring and accounting for environmental and social ‘goods and services’ (i.e. human health outcomes, ultimately) has a long history, and there has been a surge in social valuation work in the UK following recent initiatives in this area, including the growing recognition of the limits of GDP and a consumption-based growth model; the establishment in 2010 of a new national measurement of wellbeing by the Office for National Statistics; and the Public Services (Social Value) Act 2012 [11–16]. That said, these interviews suggest there is both scepticism as to the efficacy of this kind of legislation and that social valuation methods are being used by some private sector actors primarily as marketing, and possible negotiation, tools.

There is no space within this paper to expound substantially on the potential applications and pitfalls of valuation approaches - the methods and findings from its use in this pilot are detailed in a sister paper, and ongoing research in this area forms a central part of a new large-scale follow-on research programme [4, 13, 17]. In short therefore, there are multiple limitations to consider, including:

- Limits to quantification: there is a long history of detraction of a quantified approach to accounting for social and environmental (health) outcomes, both outside and within the environmental world and for a range of reasons, from issues of practicality (i.e. measuring the unmeasurable; ignoring wider political forces) to fundamental moral values (i.e. how can we put a price on fresh air?) These concerns echo Oscar Wilde’s oft-cited description from his 1892 play, Lady Windermere’s Fan, of a cynic as ‘a man who knows the price of everything and the value of nothing’ [18–22].
- Limits in conceptualisation: there are long-standing debates too around the meaning, understanding and subjective nature of worth and value (e.g. Rubbish Theory: ‘one man’s trash is another man’s treasure’; the re-evaluation of waste in circular economies). ‘The vain quest, as some would see it, to place a monetary value on materials in the wrong place (as ‘pollutants’), should be put aside in favour of the more rewarding challenge of recognizing the Rembrandt print in the junk-shop window’. [23–26]
- Limits in governance: Approaches to valuation are relatively powerless in the face of national and international models of governance, political ideologies and core values (i.e. values trump valuation in decisions relating to, for example: national infrastructure investment; targeting of financial incentives; the role of competition) [27].

The various challenges are usefully summed up by Colin Mayer in his 2013 book, ‘Firm Commitment: Why the corporation is failing and how to restore trust in it’:

‘…attempts to determine social value have been less successful…Determining likely future impacts is hard, attaching values to them is still harder, and evaluating the appropriate rate at which to discount the future costs back to the present is well nigh impossible.’ [28]

However, Mayer goes on to acknowledge that ‘our failure to account for the depreciation of the world’s stock of natural capital is having devastating consequences for our well-being and the survival of our descendants’, and he proposes an alternative way forward where values are ‘not defined by unreliable and subjective discounted future environmental costs, but by the lowest costs at which any level of pollution can be avoided.’ [28] The subject of valuation is tackled far more comprehensively by his colleague and fellow Natural Capital Committee member, Dieter Helm, who argues: ‘…knowing where we are, and what is really going on in our economy, is a necessary step to meet the challenge of doing something about it. This needs measurement and numbers.’ [29]

Interviewees’ responses in this pilot study suggest that the ‘value of valuation’ is not necessarily in its precision, but in its ability to enable (a) an understanding of the scale of an issue, (b) a comparison between different issues and (c) subsequent prioritisation. They identified a wide range of potential intervention points where this kind of non-market valuation may be employed across the ‘system of systems’ involved in urban development (Fig. 1). [30] The caveats given by our pilot interviewees around validation and fairness underline the need both to test practical application and to investigate wider systemic considerations, including the role of governance. To effectively bridge the policy-implementation gap, newly developed methods of valuation must fit and add value to specific practitioner processes of (and tools used in) decision-making, which requires deep understanding of context and effective knowledge brokerage [31–35]. Given the complexity of these systems, unintended consequences should be explored as fully as possible [36, 37]. A clear challenge therefore is the nuance of how to take this work forward. Drawing on interviewees’ responses, Box 3 suggests areas of future investigation for next stage valuation in this space.

Box 3 Suggested follow on research questions for economic valuation

**Table:** 

• What are the most suitable mechanisms for assessing non-market valuations in this urban development context? • Who should be responsible for undertaking these valuations? If the private sector, how do public sector agencies assess its validity, and vice versa? • What scope is there for misuse? (i.e. either by supporting the case for development or, conversely, by justifying that a development shouldn’t proceed) • If viability is compromised by these valuations, does that mean development shouldn't go forward? What alternatives are there? • How does this form of valuation fit with policy and political priorities (e.g. if housing delivery is given priority over issues of planetary health) • How might central government and other agencies factor in these external costs in to their assessment mechanisms (e.g. UK Govt Green Book and RICS Red Book)?	

There has been considerable, high profile research and political debate investigating short-termism in finance and corporate governance globally, particularly following the 2008 financial crash and the increasing calls to address the climate and ecological emergencies. [28, 38–41] Though it is likely to be only a part of a much more complex system, control of finance and corporate behaviour are fundamental to urban development. Linking findings from this crucial and highly important area to urban development systems appears to be a missing and potentially vital piece of the puzzle.

With regard to finance and public-private partnership, there have been widely reported concerns in the UK about certain private finance initiatives, particularly with regard to debt repayments [42–44]. Interviewees pointed out that current dominant private development models deliver community infrastructure towards the end of the process (i.e. returns from the sale of the properties is accrued and infrastructure paid for at the end), which contravenes best practice in terms of locking in habits early, and with developer contributions often insufficient to pay for the infrastructure that’s needed [45–49]. This latter issue is contested by mainstream developers on the grounds that viability would otherwise be compromised; hence we list it above as a potentially irreconcilable tension (Boxes 1 and 2). With regard to financing of development, the May Government lifted the borrowing cap for local government shortly after we undertook these interviews (October 2018), which may go some way towards addressing this issue (Box 1), but further investigation is needed to ascertain whether this has resolved that tension [50].

The control and use of public sector land were widely seen by interviewees as crucial to securing healthy urban environments. Bearing in mind the significantly reduced capacity within the public sector currently, exploring how health might be better integrated into public sector land disposal mechanisms appears to be a clear and much needed area of investigation. It was acknowledged that a range of factors are already ‘baked in’ to land values (e.g. affordability requirements), while some externalities (e.g. air quality, noise) are factored in more generally via planning policy, but that—in the main—health is not. There was a general acknowledgement too that existing mechanisms for the identification of new land for development (e.g. via ‘Housing and Economic Land Availability Assessments’) are inadequate in assessing appropriateness of development in health terms. Land ownership and land value capture are not new areas of interest, and there is considerable debate ongoing in this area [51, 52]. That public and private sector interviewees both broadly supported land value capture is perhaps not so surprising. Major historical figures, including Adam Smith, one of the founding fathers of modern economic systems (the Adam Smith Institute describes itself as ‘Britain’s leading free market neo-liberal think tank’), and Winston Churchill, declared their distaste for unearned income. Smith famously stated that ‘landlords….love to reap where they never sowed’ in his *Wealth of Nations*, and Churchill described land in 1909 as ‘the mother of all monopolies’ [53, 54]. That said, we do not infer that *public* land ownership necessarily results in healthy urban development. There are very few truly exemplary urban developments in the UK and indeed a number of the most well-known have been developed by philanthropic private-sector industrialists, landowners and financiers: e.g. Cadbury, Duchy of Cornwall, Peabody (an interesting link here perhaps between Quaker-inspired or similar principles and the modern movement towards social purpose in corporate governance) [55–58]. The significant ownership of land by the state is in fact a relatively new phenomenon: all land in the UK is, in theory at least, still owned by the Crown and has been largely privately controlled since the time of William the Conqueror (i.e. the 11^th^ Century) [54]. The Enclosures—the prolonged displacement of people off the land and in to the cities, predominantly through acts of parliament in the 18^th^ century and onwards—are often cited in this regard but in fact were about rights of access, rather than ownership [54]. These are not just economic or ‘territorial’ issues but moral and socio-political. It is perhaps unsurprising that any proposed changes to the current status quo have led to tensions, not only given that volume house-builders now deliver 80% of new homes in the UK and that construction contributes 6.1% of GDP but also that the development of new homes is a core part of government’s strategy for addressing affordability, a perennial priority policy area. The question raised by interviewees about who pays—big US technology firms or UK landowners—flags that this is not just a local, peripheral issue, but one that is both complex and in need of national and even international consideration [59, 60].

Though partnership was reported to be of fundamental importance, the defining characteristics given – e.g. trust, track record, shared vision – were notably vague and lacking in clear definition. Possible innovations in this space appear barely conceived, which suggests a significant need for greater evaluation, innovation and dissemination in this reportedly highly important area.

With regard to politics, there is considerable literature on the links, both positive and negative, between (short-term) democracy and (long-term) sustainability, so it is perhaps remarkable that not much was mentioned about this in the interviews [61–63]. That said, this dearth of response may have multiple reasons: e.g. the composition of the pilot research team, sample group and starting interview themes. However, a key point was made about prioritization and agenda-setting: the example given was that discussions on urban development are dominated by housing numbers and affordability, followed by air pollution, with little room for anything else. This suggests there may be an opportunity to raise the broader aim of planetary health (and equality) up the agenda. If so, there is a question as to what role the fields of education, political science and other linked areas should take and what scope public and private sector decision-makers have in addressing this issue. The following statement from one of the interviewees may sound obvious, but it sums up the challenge well: ‘…if you want to embed sustainable long-term change…quietly create some sort of political consensus’.

Solving the issue of public realm—its ownership and maintenance specifically—is clearly seen as a significant challenge in both public and private sectors. That no one should want to bear the cost is unsurprising, but the lack of ready solutions was revealing and points to an urgent need for further research into potential mechanisms and innovations. An oft-repeated call during the interviews, from the private real estate and public sectors, was for estate management and stewardship, whereby revenue streams are linked to quality of privately-owned public realm. However, there are issues that need to be overcome (e.g. public access and use: ‘no ball games’) and high-quality examples appear to be constrained to high value city centres locations where demand is high and affordability an issue. The plight of the high street and its retail, and the subsequent calls for solutions, may provide lessons for ways of seeking potential innovation in this space [64].

The role of national government in the making of policy, legislation and regulation was seldom raised by interviewees, other than to question its efficacy. This was surprising and underlines a potentially urgent need to think more systematically around issues of core influence (e.g. the role of policy and legislation in other areas, such as short-termism in finance, land control and disposal). Two interesting example suggestions were given, which may provide useful cases to consider: firstly, that the built environment, given its impact on the wider population, could be regarded in law as a common asset, opening up the possibility of exploring the boundaries between public and private land and asset control and secondly, that empty buildings could be considered a ‘public bad’ (as a contrast to the widely understood term ‘public good’) and therefore command a different status under mechanisms such as Compulsory Purchase Orders.

With regard to capacity, statements from interviewees on the transfer of professional resource and expertise from public to private sector over recent years were unsurprising, but they do emphasize the significant current imbalance in the public-private relationship in the UK: a recent Royal Town Planning Institute survey reported that 74% of private sector respondents felt that they had the resources they needed to deliver their goals, as opposed to 28% in the public sector [65]. This imbalance further supports the view that the private sector currently has the lion’s share of resource and—outside the granting of planning permission and policy-making—largely controls the main mechanisms of urban development (land control, finance, delivery), so any focus for improvement in human and planetary health outcomes must therefore take this in to consideration [2, 3, 66, 67]. Given this resource imbalance, it is remarkable therefore that the prevailing view given by private sector is that initiatives on healthier urban development needed to come from the public sector.

Though presented last, this and other intractable challenges identified highlight some of the most fundamental barriers to achieving healthy urban environments (e.g. conflicting priorities, access to finance and land). Future research should therefore include these in their areas of focus when exploring possible opportunities. In situations where there are no ‘elegant’ solutions, politically or otherwise, means of brokering compromise may become paramount. In these cases, the notion from the anthropological sciences of ‘plural rationality’—identifying differing values and world views, and navigating towards compromise and ‘clumsy solutions’ (rather than consensus)—may offer useful frameworks for achieving positive outcomes in these important areas [68–70].

## Conclusion

The interviews have provided a rich insight for those seeking to integrate planetary health across complex urban planning and development systems in the UK, and we hope to have contributed further to that in the discussion. It should be highly pertinent to those involved in healthy urban planning and development globally, especially in similar market-led economies. Together they reveal the numerous, overlapping and interconnected knowledge domains involved, a very wide range of barriers and potential opportunities and the need for holistic consideration in the development of interventions. There is no silver bullet, but this pilot has enabled the identification of a number of clear routes to potential impact, not just through the validation and application of non-market economic valuation but through the testing of targeted multi-action interventions in key areas, such as agenda setting and prioritisation, land disposal considerations and valuation, financial reporting mechanisms, corporate governance assessment, best practice partnership working; national instruments outside of planning policy. public realm ownership and maintenance and education and messaging. Box 4 summarizes the headline messages and next steps.

How our urban environments are planned, developed and managed involves a bewildering array of stakeholders, contexts, histories, processes and influencing factors. Related disciplines include, by no means exclusively: planning, law, finance, risk, architecture, economics, engineering (civil, mechanical, digital, etc.), politics, surveying, landscape architecture, transport planning, community involvement…and the list goes on (hydrology, retail, climate science, social care, primary care, education, waste disposal, etc.) The narrow specialisation that dominates both academia and real-world practice today naturally precludes the holistic understanding that is necessary for grappling with this highly complex area. [71, 72]

A ray of hope perhaps may be that, while on the one hand there is so much to bring together, on the other hand many of the disciplines involved are already well established fields of practice with long-standing knowledge domains (e.g. urban planning, corporate law, risk management, political science, systems science). In other words, much of the knowledge for how to solve these challenges may well already be there: i.e. ‘we know what to do, we just lack the political will.’ This logic suggests it is more of an exercise in engagement and the bringing together of existing knowledge domains: an endeavour that is potentially both achievable and highly impactful.

A common thread from interviewees’ responses was that there is no single solution: i.e. interventions need to be composed of multiple actions across whole systems: ‘look at what the Hackett Review is recommending around fire safety… something that’s tackled at every single phase.’ It is therefore heartening to see funders call increasingly for multi-action interventions, and trans-disciplinary, impact-focused research, as recommended in the Stern 2016 Review on the Research Excellence Framework. [73] Given the extraordinary pressures at play (e.g. the climate, ecological and equality emergencies, the growing burden of non-communicable diseases), bold new exploratory research is needed, and quickly, such as those initiatives pioneered by Wellcome’s Our Planet Our Health, the Belmont Forum and JPI Europe’s Sustainable Urbanisation and Governance Initiative, and the UK Prevention Research Partnership, all of which are explicit about these challenges and new approaches needed [74–76]. It is also heartening to see the proactive engagement from practitioners and funders involved in this study, which suggests there may be considerable appetite across a wide range of sectors and communities to work together to solve these challenges.

Box 4 Summary of headline messages drawn from the interviews

**Table:** 

**1. Agenda-setting and prioritisation:** Establish what level of priority human and planetary health is given in the strategic and day to day decision-making of urban development decision-makers, at local and national level, and how priority might be elevated. **2. Balanced, comprehensive valuation:** Establish how to validate non-market economic valuation within urban development, and how to ensure a level playing field (see Box 3). **3. Short-termism and corporate governance:** Bring to bear the considerable work being undertaken recently investigating financial short-termism and corporate governance with this exploration of healthy urban development. **4. Balanced partnership:** Establish what constitutes the optimal balance in partnership—between public, private and community—including determining the right balance (of power and resource) between parties. **5. Land control:** Establish how planetary health might be integrated into public sector land disposal mechanisms. **6. Land value:** Integrate work in human and planetary health into current conversations about land value capture (including consideration of broader issues of equity and taxation). **7. Identifying ‘good’ partners:** Evaluate and develop innovations in developer models, and what being a good partner means (factoring in: agenda setting and prioritization, trust, track record, shared vision, time horizons). • **The public realm challenge:** Evaluate and develop innovative solutions to the issue of public realm ownership and maintenance (including consideration of private estate management and stewardship, versus public access and use; exemplars outside high value city centre locations). • **The role of government:** Investigate the role of national government policy, legislation and regulation in areas outside of planning policy (e.g. short-termism in finance, land control and disposal), and their potential impact on healthy urban development. • **Finding compromise:** Investigate how to resolve (or broker compromise between) irreconcilable tensions identified (Box 3)	

## Supplementary Information

ESM 1 (DOCX 50 kb)

## Abbreviations

CCG: Clinical Commissioning Group
GM: Greater Manchester
JSNA: Joint Strategic Needs Assessment
JSP/JTP: Joint Spatial/Transport Plan
LEP: Local Enterprise Partnership
MHCLG: Ministry for Housing, Communities and Local Government
NHS: National Health Service
PPP: Public Private Partnership
RICS: Royal Institute of Chartered Surveyors
WECA: West of England Combined Authority
