# Supplementary material for: Overcoming Systemic Barriers Preventing Healthy Urban Development in the UK: Main Findings from Interviewing Senior Decision-Makers During a 3-Year Planetary Health Pilot
Source: J Urban Health. 2021 May 3;98(3):415–27. doi: 10.1007/s11524-021-00537-y (PMC8190222; doi:10.1007/s11524-021-00537-y)
Supplement: Supplementary file 1 — (DOCX 50 kb) [file 11524_2021_537_MOESM1_ESM.docx]

**SUPPLEMENTARY MATERIAL**

**Overcoming systemic barriers preventing healthy urban development in the UK: main findings from interviewing senior decision-makers during a three-year planetary health pilot**^[[1]](#footnote-1)^

**Daniel Black**

Daniel Black + Associates | db+a, UK

**Paul Pilkington, Ben Williams, Janet Ige**

UWE Bristol, UK

**Emily Prestwood**

University of Birmingham, UK

**Alistair Hunt, Eleanor Eaton**

University of Bath

**Gabriel Scally**

University of Bristol, UK

The following anonymised quotations are extracts from the interview transcriptions and link to the paper above via the same eight main themes that emerged from the interviews.

**Urban development agencies’ understanding of urban health**

“*Almost surprised it’s not more. Bears out my assumptions*.”

“*All expected*.”

“*Broadly not surprising*.”

“*Most are obvious, but dementia* *surprising*”.

**Valuation**

*“…the negative impacts… how are they captured…they’re not at the moment!”*

*“I would like to see some value put against them, but ultimately...it's very difficult to value softer issues…”*

*“There’s no leap of faith here for people, I don’t think...people get it…the challenge will be actually what are the costs associated…”*

*“…I can certainly see from a sales point of view…there's some valuable evidence here that would help us.”*

*“…this looks like a really useful checklist…actual costs are less important…”*

*“…it would be more about the narrative, at the moment anyway, than the specific cost-benefit…”*

*“…the values have to be authenticated by somebody…”*

*“…will it or does it influence my thinking? Yes…depending on the assumptions that go into it…”*

**Finance**

*“…not so long ago there was a bit more about maximising stakeholder value, but it’s definitely shareholder value today.”*

*”…making a profit, but not profiteering...”*

*“…in the current model, developers build and get out...”*

*“It's hard for me to see how the business model can adapt at not being so short-term…we get measured every single six months of the year as to how well we're doing.”*

*“…there’s probably, actually, a queue of pension fund investors wanting to get in.”*

*“…some of the UK pension funds still want returns back in seven years…If you look at the Royal Docks in East London, that is a Chinese investor and they're not in it for five years, they’re in it for 20 years….”*

*“they’re more cash hungry; they turn their capital slower…to get the return on capital employed you have to make a higher profit margin.”*

*“They might be borrowing at four, five, six per cent. We can borrow at about one and a half.”*

*“…they (housing associations) are not measured the same way as us, and they have access to very cheap money…”*

**Land**

*“…rather than selling off sites’* (which was) *‘the historic way of doing it”.*

“…*there are now more joint ventures, more local authority development companies being established where the control and the value capture can be, as far as possible, maintained*”

*“…there’s an ability to sell land to the ‘short termist' at the highest price”*

*“….why aren’t (the NHS) actually parcelling up some of (their) land and saying, “with the proceeds I can get from the land sale…”*

*“…our business, a fundamental part of it is land acquisition. We can’t do anything without land…”*

*“I think land value capture is really key…there’s a cost and where’s that money coming from?”*

“…*it’s* *very* *dangerous when you try and intervene in things like (land value capture)…*”

*“…it’s very easy to say, oh well, we can take a five per cent tariff off land values…Where are the Googles? Why are the rich not paying more tax?”*

**Partnership**

*“…the key thing there is…make sure you’ve got an alignment of interest financially as well as in terms of governance and culture, that’s absolutely critical...”*

*“You’ve got to understand what you’re putting in to the pot, and be able to value that. If it’s only 10% of the partnership, then you only get 10% of the control.”*

*“…(developers) do vary enormously…There have been clients who would genuinely talk to people and those who tell people what they have decided to do already.”*

*“…private developers, you’ll get good ones and bad ones…”*

*“…the demands from (housing association) tenants are very, very, different to what they might be from private occupiers…they manage them to be functional...”*

*“…for twenty-five years I’ve been thinking that they will wipe the floor with us because they don’t have to borrow money…they’ve got such a big stock to borrow against...”*

‘*What do they do if their speculation fails? Can they increase rents?*’

**Politics, education and communication**

*“If health, air quality and noise are high up your agenda, then you’ll deliver it.”*

*“…one of those first things is raising it on a political agenda, making sure that this is seen as a priority”*

*“…the huge priorities are supply and affordability* (of housing)*…”*

*“…elections…drives a lot of extremely short-term decision-making...”*

*"Politicians benefit from announcing a policy; they don't always benefit from its delivery"*

*“…if you want to embed sustainable long-term change…quietly create some sort of political consensus.”*

**Public Realm**

*“…without some kind of public sector intervention I can’t see how the private sector can maintain all the public realm…”*

*“…we don’t retain an ownership in a development…our business is about developing and selling houses and moving on…”*

**Policy, legislation, and regulation**

*“…there was a Planning Guidance (PPG3) that set minimum densities…the thinking behind it was that if you increased densities then you would use less land or you would be able to get more houses on them…now if you visit those developments, they are a complete nightmare...”*

*“…we have already got policies in our local plan that supports sustainable transport, and yet we ask ourselves, “how come what we’re getting isn’t what we want at the end of the day?”*

*“…we’ve talked about (health assessment policy) actually, and I suppose I’m yet to have a view as to whether or not (it would) generate a lot of paper that makes no difference…”*

*“The Social Value Act strikes me as a pretty limp piece of legislation.”*

*“…we developers would do these if there was a level playing field…”*

*“If there’s no commercial imperative to do it and there’s no statutory requirement to do it, nobody’s going to do it.”*

**Capacity and Resource**

*“…what the public sector are prepared to pay to attract the right skills is limited…”*

*“…young people in the public sector ultimately get bogged down in the politics or the lack of opportunity and they move on.”*

*“Anybody that becomes any good or makes any real progress in a local planning authority tends to get snapped up by the private sector and I don't know exactly what you could do about that."*

*“There’s no doubt there’s a lack of resource in a lot of authorities…it’s not a big issue for us”*

**Acknowledgements**

All authors contributed substantially to this paper. Daniel Black led the interviews, primary analysis and reporting with support from Paul Pilkington and Gabriel Scally. Ben Williams, Janet Ige and Emily Prestwood led the interview recording, coding and parallel analysis. Figure 1 in this paper was conceived and produced by Daniel Black. The economic valuation work in UPSTREAM was undertaken by Eleanor Eaton and Alistair Hunt at the University of Bath’s Department of Economics. All members of [UPSTREAM Consortium](https://urban-health-upstream.info/team/) inputed into discussions during the project, especially Judy Orme and Laurence Carmichael, but also Jim Longhurst, Kris Ebi, Roderick Lawrence, Sophie Laggan, Ruth Larbey and Margarida Sardo. Thanks also to our external advisors – Neil Smith, George Ferguson, Yolanda Barnes, Ian Cox and Barra Mac Ruairí – for contributing their time at the start of the pilot to helping develop the initial thematic areas of enquiry. This paper has been benefitted too from broader consideration and input through the early development of the five-year TRUUD research programme ([Tackling Root Causes Upstream of Unhealthy Urban Development](https://www.bristol.ac.uk/population-health-sciences/projects/truud/)), which is funded through the [UK Prevention Research Partnership](https://ukprp.org/) and includes: the British Heart Foundation, Cancer Research UK, Chief Scientist Office of the Scottish Government Health and Social Care Directorates, Engineering and Physical Sciences Research Council, Economic and Social Research Council, Health and Social Care Research and Development Division (Welsh Government), Medical Research Council, National Institute for Health Research, Natural Environment Research Council, Public Health Agency (Northern Ireland), The Health Foundation and the Wellcome Trust. ^17^ ^81^

1. UPSTREAM was a three-year pilot funded by the Wellcome Trust under their Our Planet Our Health (OPOH) Programme, which supports researchers to take on the challenges that i) food systems, ii) increasing urbanisation and iii) climate change pose to our health. It was funded under the second round of pilot awards and sits within the urbanisation theme. [↑](#footnote-ref-1)
